# Field evaluation of a ready-to-use combined *Porcine circovirus* type 2 and *Mycoplasma hyopneumoniae* vaccine in Denmark – a historical comparison of productivity parameters in 20 nursery and 23 finishing herds

**DOI:** 10.1186/s40813-018-0104-7

**Published:** 2018-12-07

**Authors:** Gitte Blach Nielsen, John Haugegaard, Rika Jolie

**Affiliations:** 1 MSD Animal Health Nordics, Havneholmen 25, DK-1561 Copenhagen V, Denmark; 2 Merck Animal Health, 2 Giralda Farms, Madison, NJ 07940 USA

**Keywords:** PCV2, *Mycoplasma hyopneumoniae*, Vaccine efficacy, Historical comparison

## Abstract

**Background:**

In practice, field evaluation of vaccine efficacy in individual herds is often based on a historical comparison of productivity data following initiation of vaccination. Being biased by time, this study design highly contrasts the more controlled, parallel-group design used for most initial vaccine efficacy studies but offers the possibility of including a larger number of animals and herds. As an important add-on to previous findings in controlled studies, the objective of this study was to evaluate the field efficacy of the ready-to-use combination vaccine Porcilis® PCV M Hyo (MSD Animal Health) by an observational historical study design using routinely generated herd productivity data.

**Results:**

Data on mortality, average daily weight gain and feed conversion rate were collected as yearly averages for one year prior to and one year after implementation of Porcilis® PCV M Hyo vaccination from 20 nursery and 23 finishing herds. When comparing pre- and post-vaccination periods, the average improvements in productivity data amounted to − 0.4 percentage points for mortality (*p* = 0.014), + 5 g for average daily weight gain (*p* = 0.555) and − 0.06 feeding units(FU)/kg for feed conversion rate (*p* = 0.074) in nursery herds and − 0.5 percentage points for mortality (*p* = 0.012), + 34 g for average daily weight gain (*p* < 0.001) and − 0.04 FU/kg for feed conversion rate (*p* = 0.133) in finishing herds. Even though some nursery and finishing herds also previously vaccinated against PCV2 and/or *Mycoplasma hyopneumoniae*, this did not significantly affect the results. For finishers, these results were obtained when difference in arrival weights between the periods and shared ownership of the herds were additionally taken into account.

**Conclusion:**

In these 20 nursery and 23 finishing herds, previous findings from parallel-group vaccination studies concerning average daily weight gain for finishers were confirmed. Additionally, a significant effect on mortality for both nursery and finishing herds was demonstrated in this evaluation based on routinely generated herd productivity data.

**Electronic supplementary material:**

The online version of this article (10.1186/s40813-018-0104-7) contains supplementary material, which is available to authorized users.

## Background

Since the safety and efficacy of Porcilis® PCV M Hyo^1^ against the combined infection with Porcine circovirus type 2 (PCV2) and *Mycoplasma hyopneumoniae* (*M. hyopneumoniae*) was first described [1], the vaccine has been used widely. Later studies in individual herds with a parallel-group study design have demonstrated improved average daily weight gain of 30–50 g and reduced enzootic pneumonia-like lung lesions compared to non-vaccinated controls [1–3]. Some randomized, parallel-group field studies have also demonstrated superiority of Porcilis® PCV M Hyo compared to other PCV2 + *M. hyopneumoniae* vaccines concerning significantly reduced enzootic pneumonia-like lung lesions [3, 4] and PCV2 viremia [3]. No significant effect on mortality has been found in any of these studies, which could be speculated to be partly due to the PCV2 infections being subclinical (as in [1–5]) and partly due to the parallel study design allowing non-vaccinated pigs to benefit from vaccinated pigs (in terms of infectious pressure) and vice versa due to commingling [6] Usually, all pigs in a herd are either vaccinated or not, and individual-herd vaccine effect-evaluation is primarily based on whether the routinely generated herd productivity reports show improvements in mortality, average daily weight gain and/or feed conversion rate following implementation of a vaccination strategy. Although such a historical comparison has the obvious confounding effect of time, the impact of vaccination on i.e. productivity parameters is not compromised by the presence of non-vaccinated pigs, increasing the group infectious pressure. Historical comparisons using routinely generated productivity data for evaluation of a vaccine’s efficacy is therefore an important add-on to findings in parallel studies, directly reflecting the foundation for efficacy evaluation in the field. The objective of this study was to evaluate the field efficacy of Porcilis® PCV M Hyo in a larger number of herds by an observational historical study design using routinely generated data from individual herd productivity reports.

### Regional context

In Denmark, Porcilis® PCV M Hyo became commercially available in November 2014. Reasons for initiating a vaccination program against PCV2 and *M. hyopneumoniae* generally originate from a cost/benefit assessment. While this is ultimately true for finishing herds, nursery herds might also vaccinate due to a request from the purchaser of growers. In the Danish production system, the nursery period lasts from weaning at 6–7 kg to around 30 kg and the finishing period from 30 kg to around 110 kg live weight.

## Herd characteristics

Herd identification was done in cooperation with swine veterinarians in order to find herds which had been vaccinating with Porcilis® PCV M Hyo according to label during minimum 1½ years and which routinely generated herd productivity reports. Furthermore, their herd health status had to be unchanged [7] during the study period (see Additional file 1). Productivity data collection was done in two steps, first from nursery herds and secondly from finishing herds as it was soon realized that only three of the nursery herds also generated productivity data for the finishing age group (nursery herd 1 incl. Finishing herd 6, nursery herd 4 incl. Finishing herd 2 and nursery herd 11 incl. Finishing herd 1). Thus, nursery and finishing herds were analyzed separately.

All herds were positive for *M. hyopneumoniae* and, to a varying degree, other respiratory pathogens such as Porcine Reproductive and Respiratory Syndrome (PRRS) and *Actinobacillus pleuropneumoniae* (*A. pleuropneumoniae*) serotypes 2, 6 and 12 [7] (Additional file 2: Tables S1 and S2). Although the exact level of PCV2 viremia in the included herds was unknown, the infection is considered widespread in Denmark [8].

Vaccination strategies in the different herds prior to initiating Porcilis® PCV M Hyo vaccination differed substantially (Additional file 2: Tables S1 and S2), from no previous vaccination against PCV2 (8 nursery and 4 finisher herds) and/or *M. hyopneumoniae* (2 nursery and 4 finisher herds) to multiple different combinations of different PCV2 vaccines (Ingelvac CircoFLEX,^2^ Porcilis® PCV vet.^1^ and Suvaxyn PCV^3^) and/or *M. hyopneumoniae* vaccines (Ingelvac MycoFLEX^2^, Mycobac Uno Vet,^4^ Stellamune one^5^ and Thorovax^1^).

## Data collection

Study herds initiated Porcilis® PCV M Hyo vaccination during 2015 or 2016 . Data on mortality, average daily weight gain and feed conversion rate (reported as: feeding units (FU)/kg gain, where one FU = 7.38 MJ [9]) were collected as yearly averages (to avoid seasonal effects) from one year before vaccination was initiated (Period 1) to one year after vaccination was fully implemented (Period 2). All included herds used Agrosoft PigVision^6^ as the database for handling of herd data and all data for the study were derived from here. A more thorough description of data collection exists in the Additional file 1.

### Statistical analyses

Evaluation of Porcilis® PCV M Hyo vaccination efficacy was done herd-wise by calculating the change in each of the productivity parameters (mortality, average daily weight gain and feed conversion rate) by subtracting the herd average in Period 1 from the herd average in Period 2. The resulting units for the changes were therefore percentage points, g and FU/kg, respectively, and herd was the statistical unit of observation. This was done for nursery and finishing herds separately. As the changes in productivity parameters were all normally distributed, one-sample Student’s t-tests were used to determine whether the change in each parameter for nursery and finishing herds, respectively, was significantly different from zero. However, the significance level was adjusted from 0.05 to 0.017 (Bonferroni method) as three comparisons (mortality, average daily weight gain and feed conversion rate) were done for each dataset. *P*-values < 0.1 were considered tendencies.

The possible impact of previous vaccination strategies against PCV2 and *M. hyopneumoniae* as well as yearly national improvements merely due to genetics were relevant to consider for both nursery and finishing herds. As a proxy for the effect of yearly national improvement, the year of initiated vaccination with Porcilis® PCV M Hyo was used. The obtained changes were also compared to the national improvements during the same period (Additional file 2: Table S3). For finishing herds, differences in arrival weights between Period 1 and 2 as well as similarity between herds due to shared ownership were also considered.

For the change in each of the nursery productivity parameters, three linear models for each of the productivity parameters were built including previous vaccination strategy against PCV2 as a fixed effect. The variable was retained in the models at a *p*-value< 0.05. Vaccination against *M. hyopneumoniae* as well as year of initiated vaccination were not included in the linear models for nursery herds as only two herds did not previously vaccinate against *M. hyopneumoniae* and only two herds initiated vaccination in 2016 instead of 2015.

For the change in each of the finisher productivity parameters, three linear mixed models were built testing previous vaccination strategies (PCV2 and *M. hyopneumoniae*), year of initiated vaccination and difference in arrival weight for inclusion as fixed effects with owner included as random effect to account for shared ownership. Model selection consisted of a standard stepwise selection procedure based on AIC (Akaike’s Information Criterion) where fixed effects with a *p*-value< 0.05 were retained in the models. Assessment of residuals for normality was done visually. All statistical analyses were done in R [10].

## Results

### Nursery herds

Data from 20 nursery herds were included in the analyses. The average nursery herd size was 33,000 pigs produced per year (range: 10000 to 100,000 pigs). All of these provided data on mortality, 19 herds on average daily weight gain and 17 herds on feed conversion rate.

**Fig. 1:**
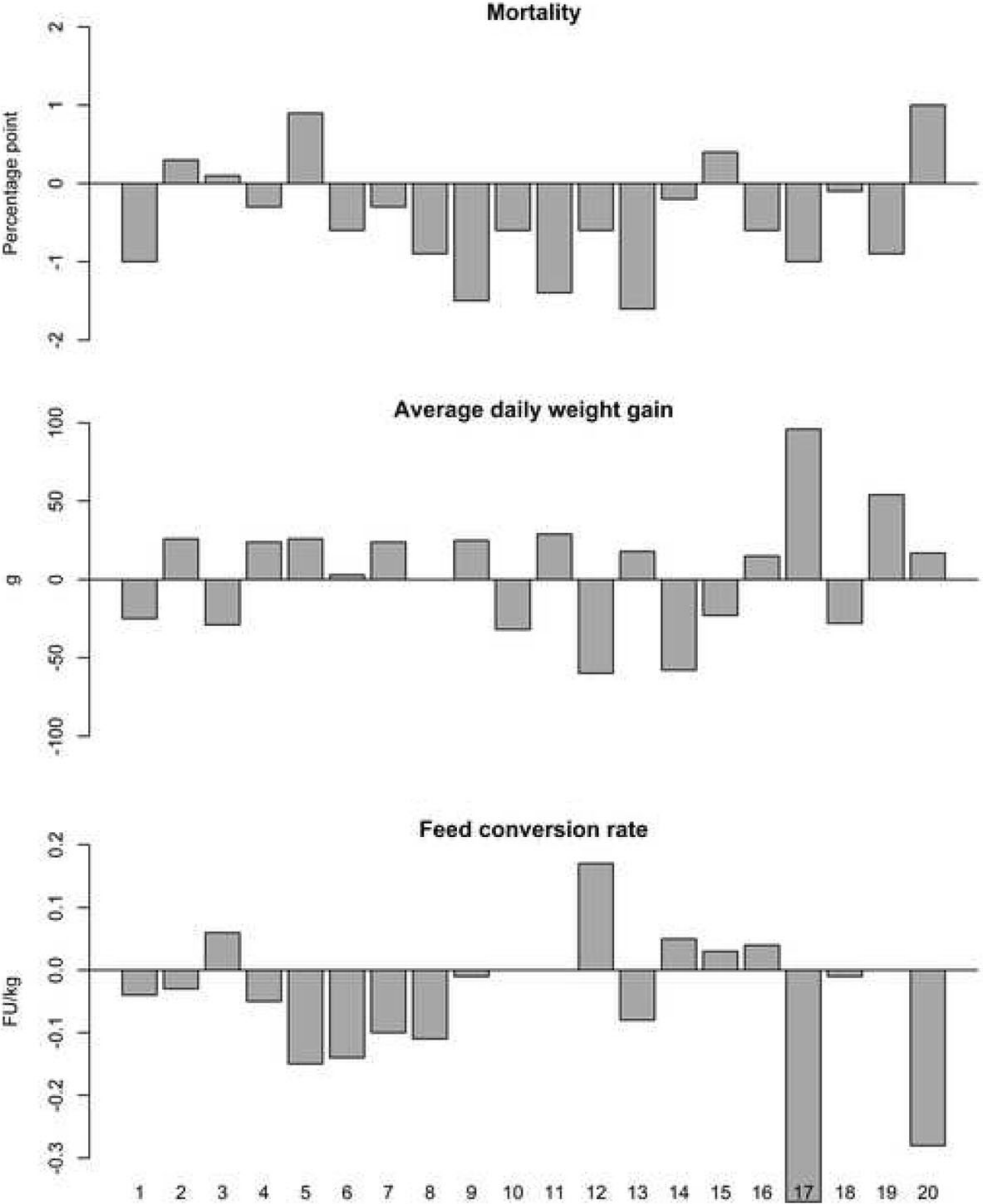
Changes for the 20 individual nursery herds in mortality, average daily weight gain and feed conversion rate between Period 1 and Period 2

Table 1 shows summary statistics concerning nursery productivity parameters for Period 1 and Period 2, and Fig. 1 displays the changes in parameters from Period 1 to Period 2 at individual herd level. On average, mortality was significantly reduced by 0.4 percentage points, average daily weight gain increased non-significantly with 5 g and a tendency for reduced feed conversion rate was seen, amounting to 0.06 FU/kg.

**Table 1:** Summary statistics concerning productivity parameters in the 20 nursery herds comparing one year before (Period 1) and one year after (Period 2) initiated vaccination with Porcilis® PCV M Hyo

Parameter	Period	Mean	Standard deviation	Median	Min	Max	*p*-value
Mortality (%)	Period 1	3.1	0.96	3.2	1.5	4.8	0.014
	Period 2	2.7	0.75	2.8	1.4	3.9	
Average daily weight gain (g)	Period 1	444	52	445	319	539	0.555
	Period 2	449	50	455	363	563	
Feed conversion rate (FU/kg)	Period 1	1.87	0.19	1.81	1.65	2.37	0.074
	Period 2	1.81	0.15	1.80	1.55	2.23

In the linear models for nursery herds, previous vaccination strategy against PCV2 was non-significant for all three productivity parameters (*p* = 0.168 (mortality), *p* = 0.286 (average daily weight gain), *p* = 0.793 (feed conversion rate)). At a national level, productivity parameters during the same period (2014 compared to 2016) increased 0.2 percentage points for mortality and 2 g for average daily weight gain, whereas feed conversion rate decreased by 0.04 FU/kg (Additional file 2: Table S3).

### Finishing herds

Twenty-three finishing herds were included in the analyses, all with a full data set regarding the desired productivity parameters. The finishing herd size ranged between 2400 and 16,000 finishers produced per year with an average of 8000 finishers.

**Fig. 2:**
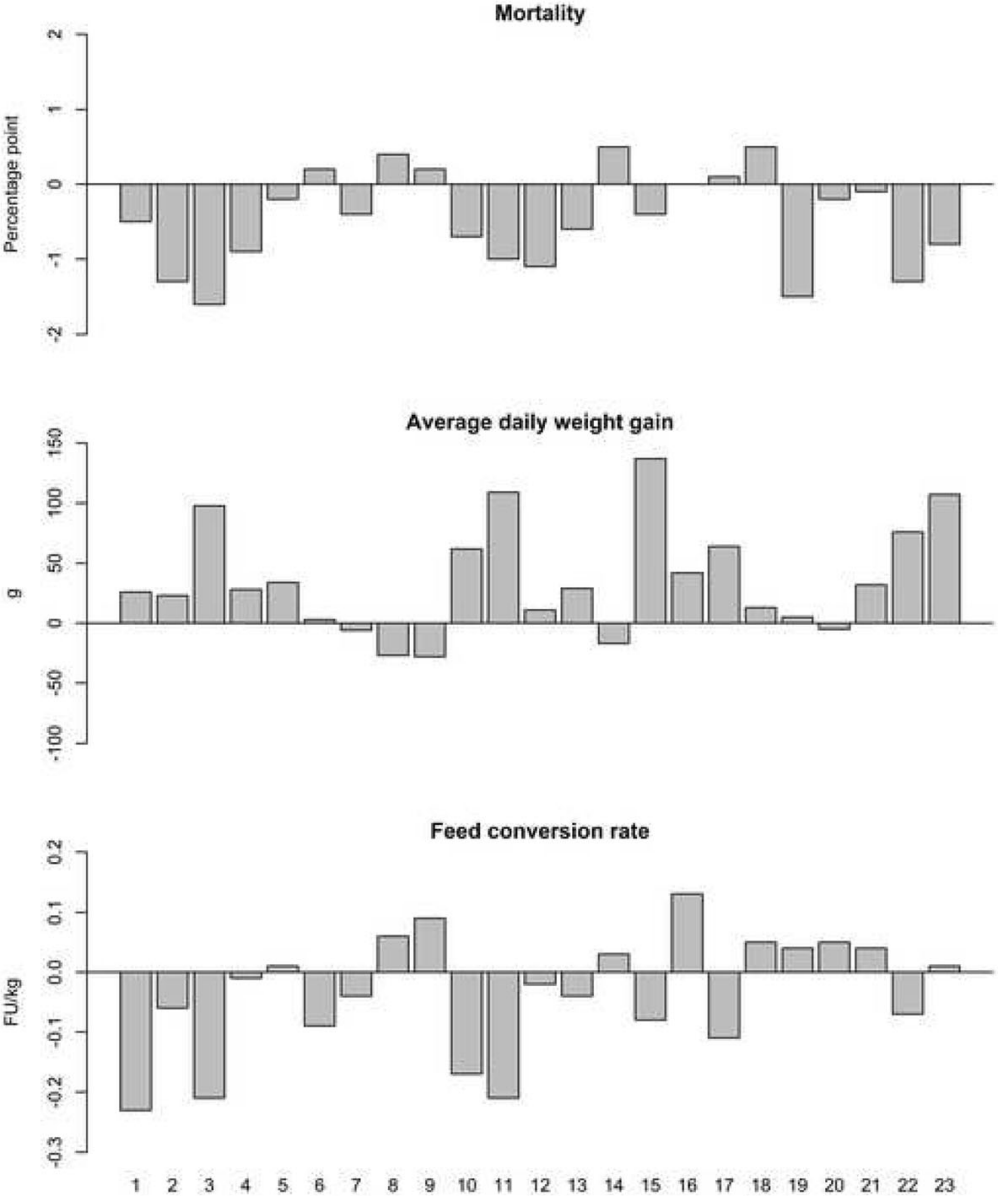
Changes for the 23 individual finishing herds in mortality, average daily weight gain and feed conversion rate between Period 1 and Period 2

Table 2 shows summary statistics concerning the finisher productivity parameters for Period 1 and Period 2, and Fig. 2 displays the change between Period 1 and Period 2 in the parameters at individual herd level. On average, mortality was significantly reduced by 0.5 percentage points, average daily weight gain significantly increased by 36 g and feed conversion rate had a statistical tendency of being reduced by 0.03 FU/kg.

**Table 2:** Summary statistics concerning productivity parameters in the 23 finishing herds comparing one year before (Period 1) and one year after (Period 2) initiated vaccination with Porcilis® PCV M Hyo

Parameter	Period	Mean	Standard deviation	Median	Min	Max	*p*-value
Mortality (%)	Period 1	3.1	1.1	2.8	1.5	6.8	0.002
	Period 2	2.6	1.1	2.2	1.0	5.3	
Average daily weight gain (g)	Period 1	970	68	976	857	1112	0.001
	Period 2	1006	64	1008	899	1153	
Feed conversion rate (FU/kg)	Period 1	2.76	0.12	2.75	2.57	3.04	0.095
	Period 2	2.73	0.09	2.69	2.58	2.98

Results from the linear mixed models are displayed in Tables 3, 4 and 5. When the effect of ownership (and difference in arrival weights for average daily weight gain) was taken into account, the changes in productivity parameters following initiation of Porcilis® PCV M Hyo vaccination amounted to − 0.5 percentage points mortality (*p* = 0.012), + 34 g average daily weight gain (*p* < 0.001) and − 0.04 FU/kg feed conversion rate (*p* = 0.133). None of the variables explaining earlier vaccination strategy (against PCV2 or *M. hyopneumoniae*) or year of initiated vaccination were retained in the final models for any of the finishing herd productivity parameters. Neither did the difference in arrival weights significantly impact the changes in mortality or feed conversion rate.

**Table 3:** Linear mixed model results regarding change in mortality for finishing herds

Parameter	Linear mixed model for change in finisher herd mortality	
	Estimates (95% C.I.)	Significance (*p*-value)
Intercept	−0.495 (−0.834;-0.169)	0.012
Fixed effects:	N/A	
Random effect:	Variance	
Owner	0.153	
Residual	0.271

**Table 4:** Linear mixed model results regarding change in average daily weight gain for finishing herds

Parameter	Linear mixed model for change in finisher herd average daily weight gain	
	Estimates (95% C.I.)	Significance (*p*-value)
Intercept	34.376 (17.763;50.988)	< 0.001
Fixed effect:		
Difference in arrival weight	7.245 (1.647;12.843)	0.020
Random effect:	Variance	
Owner	< 0.0001	
Residual	1658

**Table 5:** Linear mixed model results regarding change in feed conversion rate for finishing herds

Parameter	Linear mixed model for change in finisher herd feed conversion rate	
	Estimates (95% C.I.)	Significance (*p*-value)
Intercept	−0.037 (−0.083;0.008)	0.133
Fixed effects:	N/A	
Random effect:	Variance	
Owner	0.001	
Residual	0.009	

If the national changes in finisher productivity parameters from 2014 to 2017 (Additional file 2: Table S3) are weighted according to the distribution of specific years for individual herds for comparison between Period 1 and Period 2 in the present study, the corresponding national improvements would amount to a reduction in mortality of 0.3 percentage points, an increase in average daily weight gain of 14 g and a reduction in feed conversion rate of 0.012 FU/kg. As for the 20 nursery herds, the productivity parameter improvements seen in these 23 finishing herds numerically exceeded the national improvements during the same period.

## Conclusion

This historical comparison of productivity data for 20 nursery and 23 finishing herds following initiation of Porcilis® PCV M Hyo vaccination demonstrated a significant improvement in average daily weight gain amounting to 34 g in finishing herds, confirming previous findings in randomized, controlled trials [1–5]. In addition, a significant reduction in mortality of 0.4–0.5 percentage points was found both in nursery and finishing herds. For nursery herds, a statistical tendency towards a reduced feed conversion rate by 0.06 FU/kg (*p* = 0.07) were also found. When evaluating the size of the productivity changes, it is important to note that some of these also previously vaccinated against PCV2 and/or *M. hyopneumoniae* and that the improvements in productivity parameters for both nursery and finishing herds exceeded the improvements due to genetics in the same period. However, as data were collected retrospectively, the level of PCV2 and *M. hyopneumoniae* infections prior to initiation of Porcilis® PCV M Hyo vaccination was unknown, which is a possible confounder. Furthermore, due to the absence of non-vaccinated control herds, the study results cannot be considered directly applicable to herds in general. In future studies, a pre-vaccination diagnostic work-up in the herds would be valuable for later interpretation of results.

## Additional files

Additional file 1: Description of data collection regarding definition of Period 1 and 2, herd health status, previous vaccination status and productivity data. (DOCX 32 kb)

Additional file 2: Overview of nursery and finishing herd details and national developments in productivity data during the study period [11]. (DOCX 45 kb)

## Abbreviations

AIC: Akaike’s Information Criterion
C.I.: Confidence interval
FU: Feeding units
g: gram
kg: kilo
*M. hyopneumoniae*: *Mycoplasma hyopneumoniae*
PCV2: Porcine circovirus type 2
PRRS: Porcine Reproductive and Respiratory Syndrome

## Footnotes

1 Produced by Intervet International B.V, Wim de Körverstraat 35, 5831 AN Boxmeer, The Netherlands;

2 Produced by Boehringer Ingelheim Vetmedica GmbH, 55,216 Ingelheim/Rhein, Germany;

3 Produced by Zoetis Belgium SA, Rue Laid Burniat 1, 1348 Louvain-la-Neuve, Belgium;

4 Produced by Salfarm Danmark A/S, Fabriksvej 21, DK-6000 Kolding;

5 Produced by Eli Lilly & Company Ltd., Elanco Animal Health, Lilly House, Priestley Road, Basingstoke, Hampshire, RG24 9NL;

6 Agrosoft PigVision. Herd recording and management software. Webpage: http://www.agrosoftltd.com/. Accessed 18th May 2018.

## References

[1] Witvliet M, Holtslag H, Nell T, Segers T, Fachinger V (2015). Efficacy and safety of a combined porcine circovirus and *Mycoplasma hyopneumoniae* vaccine in finishing pigs. Trials in Vaccinology.

[2] Tzika ED, Tassis PD, Koulialis D, Papatsiros VG, Nell T, Brellou G, Tsakmakidis I. Field efficacy study of a novel ready-to-use vaccine against mycoplasma hyopneumoniae and porcine circovirus type 2 in a Greek farm. Porcine Health Manag. 2015. 10.1186/s40813-015-0006-x. 10.1186/s40813-015-0006-x PMC5382375 28405421

[3] Kaalberg L, Geurts V, Jolie R. A field efficacy and safety trial in the Netherlands in pigs vaccinated at 3 weeks of age with a ready-to-use porcine circovirus type 2 and mycoplasma hyopneumoniae combined vaccine. Porcine Health Manag. 2017. 10.1186/s40813-017-0070-5. 10.1186/s40813-017-0070-5 PMC5679184 29152324

[4] Pagot E, Rigaut M, Roudaut D, Panzavolta L, Jolie R, Duivon D. Field efficacy of Porcilis® PCV M Hyo versus a licensed commercially available vaccine and placebo in the prevention of PRDC in pigs on a French farm: a randomized controlled trial. Porcine Health Manag. 2017. 10.1186/s40813-016-0051-0. 10.1186/s40813-016-0051-0 PMC5382521 28405459

[5] Duivon D, Corrége I, Hémonic A, Rigaut M, Roudaut D, Jolie R. Field evaluation of piglet vaccination with a *Mycoplasma hyopneumoniae* bacterin as compared to a ready-to-use product including porcine circovirus 2 and *M. hyopneumoniae* in a conventional French farrow-to-finish farm. Porcine Health Manag. 2018. 10.1186/s40813-017-0077-y. 10.1186/s40813-017-0077-y PMC5772722 29375890

[6] Kristensen CS, Baadsgaard NP, Toft N (2011). A meta-analysis comparing the effect of PCV2 vaccines on average daily weight gain and mortality rate in pigs from weaning to slaughter. Prev Vet Med.

[7] Danish SPF system database, Health Status Management, Agro Food Park 15, 8200 Aarhus N, Denmark Webpage http://spfsus.dk/.

[8] Kristensen CS, Larsen LE, Hjulsager CK. In Danish: Undersøgelse af PCV2-status i to danske besætninger – to års opfølgning. Videncenter for svineproduktion. 2012;933 http://svineproduktion.dk/Publikationer/Kilder/lu_medd/2012/933.aspx. Accessed 12 June 2018.

[9] Tybirk P, Strathe AB, Vils E, Sloth NM, Boisen S (2006). In Danish: Det danske fodervurderingssystem til svinefoder. Dokumentationsrapport for energi- og proteinvurderingssystem gældende fra 1. september 2006. Dansk Svineproduktion & Danmarks JordbrugsForskning.

[10] R Core Team. R: a language and environment for statistical computing. R Foundation for statistical computing, Vienna, Austria. 2018. URL https://www.R-project.org/.

[11] Hansen C (2018). Danish: Produktivitet i svineproduktionen 2017. SEGES Svineproduktion, Notat nr. 1819.

