# Supplementary material for: Field evaluation of a ready-to-use combined Porcine circovirus type 2 and Mycoplasma hyopneumoniae vaccine in Denmark – a historical comparison of productivity parameters in 20 nursery and 23 finishing herds
Source: Porcine Health Manag. 2018 Dec 7;4:29. doi: 10.1186/s40813-018-0104-7 (PMC6284288; doi:10.1186/s40813-018-0104-7)
Supplement: Supplementary file 1 — Description of data collection regarding definition of Period 1 and 2, herd health status, previous vaccination status and productivity data. (DOCX 32 kb) [file 40813_2018_104_MOESM1_ESM.docx]

Additional file 1

## to ‘Field evaluation of a ready-to-use combined Porcine circovirus type 2 and *Mycoplasma hyopneumoniae* vaccine in Denmark – a historical comparison of productivity parameters in 20 nursery and 23 finishing herds’

Data collection – definition of Period 1 and 2

As the study was done retrospectively with a herd inclusion criterion of having minimum 1½ years of Porcilis® PCV M Hyo vaccination; herds included in the study initiated vaccination during 2015 or 2016. Depending on the specific time point of first vaccination with Porcilis® PCV M Hyo, Period 1 was calculated as one year prior to the quarter of first vaccination, i.e. if vaccination in a herd was initiated in Q4 2015, Period 1 lasted from Q4 2014 to Q3 2015 (both included). Because piglet vaccinations against PCV2 and *Mycoplasma hyopneumoniae* in Denmark generally occur around weaning (corresponding to 3-6 weeks of age), productivity data of the six months immediately following first vaccination were excluded because, during this period, routinely generated data were derived also from non-Porcilis® PCV M Hyo-vaccinated pigs. Data considered to be derived only from Porcilis® PCV M Hyo-vaccinated pigs was therefore collected from six months after the first vaccination and one year onwards. I.e., if vaccination in a herd was initiated in Q4 2015, Period 2 lasted from Q3 2016 to Q2 2017 (both included). This protocol was used for all study herds to determine Period 1 and 2.

Data collection – herd health status

Definition of herd health status was based on serology. As herds in the Danish SPF (specific pathogen free) system [7] are blood sampled yearly to confirm their serological status concerning *Mycoplasma hyopneumoniae*, *Actinobacillus pleuropneumoniae* serotype 2, 6 and 12 and Porcine Reproductive and Respiratory Syndrome (PRRS), the definition of herd health status was based on this official SPF status. As part of this system, additionally, clinical surveillance of these diseases as well as lice, mange, swine dysentery and atrophic rhinitis is performed by the herd veterinarian. In a herd, where none of these diseases/pathogens are present, the herd health status would be ‘SPF’. In a herd only being infected with *Mycoplasma hyopneumoniae*, the herd health status would be ‘SPF+Myc’, in a herd being infected with *Mycoplasma hyopneumoniae* and swine dysentery, the herd health status would be ‘SPF+Myc+Dys’ etc. Hence, herds that experienced changes in their herd health status, such as introduction or clearance of PRRS, from the beginning of Period 1 to the end of Period 2 were excluded from the study. Considering the whole 2½-year study period, this was the case for 1 nursery and 3 finishing herds [7], which were excluded from the study prior to data analysis. Herd health status of the study herds are displayed in Additional file 2: Tables S1+S2.

Data collection – definition of previous vaccination status

Herds were categorized as previously vaccinating or not according to their use of vaccines against *Mycoplasma hyopneumoniae* and/or PCV2 during Period 1 and six months prior to Period 1 based on the argumentation mentioned in section ‘Data collection – definition of Period 1 and 2’ concerning congruence between vaccination status and data.

Data collection – productivity data

Productivity data from all included herds were derived from the Agrosoft PigVision^f^ software system. Agrosoft Pigvision^f^ is a widely used system for record keeping, calculation of productivity data and productivity data analysis at herd level. As principle for nursery and finishing pigs, each entry and exit of pigs into the herd are registered continuously by date, number of pigs and total weight of these. This applies also for pigs that die. If the herd contains both nursery and finishing pigs, these registrations are done separately by age group. Similarly, the amount of feed purchased to nursery and finishing pigs, respectively, are registered by date and amount. All registrations are done by the owner/herd responsible. Then, when the farmer wants a calculation of his productivity data (i.e. average daily weight gain, feed conversion rate and mortality), he enters a ‘present total’, also by age group, of number of pigs in the herd, average weight of these and amount of feed left in the feed storage. As the last ‘present total’ is known as well as the entries and exits of pigs and feed during the intermediate time period, the system can: 1) Sum up the number of deaths in the period to calculate mortality, 2) Sum up the total number of kilos produced divided by the total number of days in the intermediate period (calculated as the sum of individual pigs´ days in the period) to calculate average daily weight gain, and 3) Sum up the total amount of feed fed during the intermediate period divided by the total number of kilos produced to calculate feed conversion rate. To adjust for differences in feed energy level in different feed mixes, the feed amount is calculated as feeding units (FU), where one FU equals 7.38 MJ [9], hence, the unit for feed conversion rate is FU/kg weight gain.

In general, calculation of productivity data in nursery and finishing age groups are done quarterly and, hence, the productivity data derived from the Agrosoft PigVision^f^ software system do not represent specific batches of pigs, rather a time period. This is well-suited for the Danish production system, where each herd often contains pigs from several batches arriving weekly or bi-weekly.
