# Supplementary material for: Field evaluation of a ready-to-use combined Porcine circovirus type 2 and Mycoplasma hyopneumoniae vaccine in Denmark – a historical comparison of productivity parameters in 20 nursery and 23 finishing herds
Source: Porcine Health Manag. 2018 Dec 7;4:29. doi: 10.1186/s40813-018-0104-7 (PMC6284288; doi:10.1186/s40813-018-0104-7)
Supplement: Supplementary file 2 — Overview of nursery and finishing herd details and national developments in productivity data during the study period [11]. (DOCX 45 kb) [file 40813_2018_104_MOESM2_ESM.docx]

Table S1: Overview of nursery herds displaying herd health status according to the Danish SPF system [7], previous vaccination strategy against PCV2 and *Mycoplasma hyopneumoniae* and yearly quarter of first vaccination with Porcilis® PCV M Hyo. Please see additional information concerning definitions of these in the Additional file 1.

| **Nursery herd** | **Herd health status**  **(Danish SPF system)** | **Previously vaccinated against PCV2** | **Previously vaccinated against *M. hyopneumoniae*** | **Quarter of first Porcilis® PCV M Hyo vaccination** |
| --- | --- | --- | --- | --- |
| 1 | Myc+Ap6+Ap12+ PRRS2 | Yes | Yes | Q2 2015 |
| 2 | Myc+Ap12 | Yes | Yes | Q4 2015 |
| 3 | Myc+Ap2+Ap12 | Yes | Yes | Q2 2015 |
| 4 | Myc+Ap2+Ap6+Ap12+ PRRS1+ PRRS2 | Yes | Yes | Q1 2015 |
| 5 | Myc+Ap6+Ap12 | Yes | Yes | Q2 2015 |
| 6 | Myc+Ap6+Ap12+ PRRS1 | No | Yes | Q2 2015 |
| 7 | Myc+Ap6+Ap12+ PRRS1 | No | Yes | Q2 2015 |
| 8 | Myc+Ap6+Ap12+ PRRS1 | No | Yes | Q2 2015 |
| 9 | Myc | No | Yes | Q2 2015 |
| 10 | Myc+PRRS2 | Yes | No | Q2 2015 |
| 11 | Myc+PRRS1 | No | No | Q2 2015 |
| 12 | Myc+Ap2 | Yes | Yes | Q1 2016 |
| 13 | Myc+ PRRS2 | Yes | Yes | Q2 2015 |
| 14 | Myc | Yes | Yes | Q3 2015 |
| 15 | Myc+Ap12 | No | Yes | Q4 2015 |
| 16 | Myc+ PRRS2 | No | Yes | Q4 2015 |
| 17 | Myc+PRRS1 | Yes | Yes | Q2 2015 |
| 18 | Myc | Yes | Yes | Q2 2015 |
| 19 | Myc+Ap12 | No | Yes | Q1 2015 |
| 20 | Myc+Ap12 | Yes | Yes | Q2 2016 |

Myc=*Mycoplasma hyopneumoniae*; Ap2=*Actinobacillus pleuropneumoniae*, serotype 2; Ap6=*Actinobacillus pleuropneumoniae*, serotype 6; Ap12=*Actinobacillus pleuropneumoniae*, serotype 12; PRRS1=Porcine Reproductive and Respiratory Syndrome, genotype 1; PRRS2=Porcine Reproductive and Respiratory Syndrome, genotype 2

Table S2: Overview of finishing herds displaying herd health status according to the Danish SPF system [7], previous vaccination strategy against PCV2 and *Mycoplasma hyopneumoniae* and yearly quarter of first vaccination with Porcilis® PCV M Hyo. Please see additional information concerning definitions of these in the Additional file 1.

| **Finishing herd** | **Herd health status**  **(Danish SPF system)** | **Previously vaccinated against PCV2** | **Previously vaccinated against *M. hyopneumoniae*** | **Quarter of first Porcilis® PCV M Hyo vaccination** |
| --- | --- | --- | --- | --- |
| 1 | Myc+PRRS1 | No | No | Q2 2015 |
| 2 | Myc+Ap2+Ap6+Ap12+ PRRS1+ PRRS2 | Yes | Yes | Q1 2015 |
| 3 | Myc | Yes | Yes | Q2 2015 |
| 4 | Myc | Yes | Yes | Q2 2015 |
| 5 | Myc | Yes | Yes | Q2 2015 |
| 6 | Myc+Ap6+Ap12+ PRRS2 | Yes | Yes | Q2 2015 |
| 7 | Myc | No | Yes | Q2 2015 |
| 8 | Myc | No | No | Q3 2016 |
| 9 | Myc | No | No | Q3 2016 |
| 10 | Myc+Ap6+Ap12+ PRRS2 | Yes | Yes | Q2 2016 |
| 11 | Myc+Ap6+Ap12+ PRRS2 | Yes | Yes | Q2 2016 |
| 12 | Myc+Ap6+Ap12+ PRRS2 | Yes | Yes | Q2 2016 |
| 13 | Myc + Ap12 | Yes | Yes | Q3 2016 |
| 14 | Myc + Ap 12 | Yes | Yes | Q3 2016 |
| 15 | Myc+Ap2+ PRRS1+ PRRS2 | Yes | Yes | Q2 2015 |
| 16 | Myc+Ap2+ PRRS1+ PRRS2 | Yes | Yes | Q2 2015 |
| 17 | Myc+Ap2+ PRRS1+ PRRS2 | Yes | Yes | Q2 2015 |
| 18 | Myc+Ap2+ PRRS1+ PRRS2 | Yes | Yes | Q2 2015 |
| 19 | Myc+Ap2+Ap6+Ap12 | Yes | No | Q1 2015 |
| 20 | Myc+ PRRS1 | Yes | Yes | Q2 2015 |
| 21 | Myc+ PRRS1 | Yes | Yes | Q2 2015 |
| 22 | Myc+ PRRS1 | Yes | Yes | Q2 2015 |
| 23 | Myc+Ap2+PRRS2 | Yes | Yes | Q3 2015 |

Myc=*Mycoplasma hyopneumoniae*; Ap2=*Actinobacillus pleuropneumoniae*, serotype 2; Ap6=*Actinobacillus pleuropneumoniae*, serotype 6; Ap12=*Actinobacillus pleuropneumoniae*, serotype 12; PRRS1=Porcine Reproductive and Respiratory Syndrome, genotype 1; PRRS2=Porcine Reproductive and Respiratory Syndrome, genotype 2

Table S3: Development from 2014 to 2017 of Danish productivity parameters for nursery and finishing herds at a national level. Modified from Table 3 and Table 4 in [11].

| **Nursery herds** | **2014** | **2015** | **2016** | **2017** |
| --- | --- | --- | --- | --- |
| Mortality (%) | 2.9 | 3.1 | 3.1 | 3.1 |
| Average daily weight gain (g) | 446 | 444 | 444 | 452 |
| Feed conversion rate (FU/kg) | 1.93 | 1.88 | 1.89 | 1.88 |
| **Finishing herds** |  |  |  |  |
| Mortality (%) | 3.7 | 3.7 | 3.4 | 3.1 |
| Average daily weight gain (g) | 931 | 947 | 950 | 971 |
| Feed conversion rate (FU/kg) | 2.84 | 2.80 | 2.81 | 2.79 |
